# Purification of a Low Molecular Weight Fucoidan for SPECT Molecular Imaging of Myocardial Infarction

**DOI:** 10.3390/md12094851

**Published:** 2014-09-23

**Authors:** Pierre Saboural, Frédéric Chaubet, Francois Rouzet, Faisal Al-Shoukr, Rana Ben Azzouna, Nadia Bouchemal, Luc Picton, Liliane Louedec, Murielle Maire, Lydia Rolland, Guy Potier, Dominique Le Guludec, Didier Letourneur, Cédric Chauvierre

**Affiliations:** 1 Inserm, U1148, LVTS, Paris Diderot University, Bichat-Claude Bernard Hospital, F-75877, Paris, France; E-Mails: pierre.saboural@univ-paris13.fr (P.S.); frederic.chaubet@univ-paris13.fr (F.C.); francois.rouzet@bch.aphp.fr (F.R.); faisal1977@hotmail.fr (F.A.-S.); rana.ben-azzouna@bch.aphp.fr (R.B.A.); liliane.louedec@inserm.fr (L.L.); murielle.maire@univ-paris13.fr (M.M.); dominique.leguludec@bch.aphp.fr (D.L.G.); didier.letourneur@inserm.fr (D.L.); 2 Galilée Institute, Paris 13 University, Sorbonne Paris Cité, F-93430, Villetaneuse, France; 3 Multimodal Imaging Research Federation (FRIM), Paris Diderot University, F-75877, Paris, France; 4 Nuclear Medicine Department, Bichat-Claude Bernard Hospital, AP-HP, F-75877, Paris, France; 5 Laboratory CSPBAT, Paris 13 University, Sorbonne Paris Cité, CNRS UMR 7244, SBMB team, F-93017, Bobigny, France; E-Mail: nbouchemal@smbh.univ-paris13.fr; 6 Laboratory of Polymers Biopolymers Surfaces, Normandie University, Rouen University, F-76821, Mont Saint Aignan, France; E-Mail: luc.picton@univ-rouen.fr; 7 Laboratory of Polymers Biopolymers Surfaces, CNRS, UMR 6270 and FR3038, F-76821, Mont Saint Aignan, France; 8 Algues & Mer, Kernigou, F-29242, Ouessant, France; E-Mails: lydia.rolland@algues-et-mer.com (L.R.); guy.potier@algues-et-mer.com (G.P.)

**Keywords:** fucoidan, molecular imaging, atherothrombosis, SPECT

## Abstract

Fucoidans constitute a large family of sulfated polysaccharides with several biochemical properties. A commercial fucoidan from brown algae, containing low molecular weight polysaccharidic species constituted of l-fucose, uronic acids and sulfate groups, was simply treated here with calcium acetate solution. This treatment led to a purified fraction with a yield of 45%. The physicochemical characterizations of the purified fucoidan using colorimetric assay, MALLS, dRI, FT-IR, NMR, exhibited molecular weight distributions and chemical profiles similar for both fucoidans whereas the sulfate and l-fucose contents increased by 16% and 71%, respectively. The biodistribution study in rat of both compounds labeled with ^99m^Tc evidenced a predominant renal elimination of the purified fucoidan, but the crude fucoidan was mainly retained in liver and spleen. In rat myocardial ischemia-reperfusion, we then demonstrated the better efficiency of the purified fucoidan. This purified sulfated polysaccharide appears promising for the development of molecular imaging in acute coronary syndrome.

## 1. Introduction

Cardiovascular diseases (CVD) are the leading cause of death in the world [1] and medical imaging is the most widespread and efficient tool for their diagnosis. Molecular imaging is a recent and promising development of medical imaging dedicated to the visualization of the biological processes *in vivo*. However, the identification of mechanisms at cellular and molecular levels relies on the development of imaging probes that are able to interact with the targeted areas. The biospecificity of these probes is achieved by conjugation with small molecules [2,3], polymers and oligomers [4,5], antibodies, proteins and peptides [6,7], polysaccharides [8,9] or mixed compounds [10]. Fucoidans constitute a family of natural sulfated polysaccharides which have been extensively studied as they exhibit numerous biological activities: anticoagulant, antithrombotic [11], anti-inflammatory [12], anti-angiogenic [13] and anti-tumoral [14], anti-complementary [15,16] and anti-viral [12]. Recently, in purified systems and with human whole blood experiments, a low molecular weight fucoidan from brown algae was found as the most efficient glycosidic ligand of P-selectin, a glycoprotein expressed on the surface of activated platelets and on activated vascular endothelium [17]. This low molecular weight fucoidan was established as a relevant targeting agent for molecular imaging of atherothrombosis, either radiolabeled with technetium-99m (^99m^Tc) for Single Photon Emission Computed Tomography (SPECT) imaging [18] or bound to Ultrasmall Superparamagnetic Iron Oxide particles (USPIO) for Magnetic Resonance Imaging (MRI) [19].

Beyond the complete determination of the structures responsible for the interaction of fucoidan with P-selectin, the development of a biospecific probe for clinical molecular imaging of CVD is required to assess the overall fate of fucoidan within tissues and organs. Due to their natural origin and non-standardized methods of purification, fucoidans form a complex family of polysaccharides varying in molecular weight, structure and composition. In order to minimize this natural heterogeneity, previous studies from our laboratory have been performed with the same commercial fucoidan (Ascophyscient^®^) provided by Algues and Mer [17,18,19,20]. This work presents a new specific treatment of fucoidan with calcium ions to obtain a purified extract. The molecular weight, structure and composition of the purified fucoidan were determined by physico-chemical techniques such as High Performance Size Exclusion Chromatography (HPSEC), Multi-Angle Laser Light Scattering (MALLS), viscosimetry, differential Refractive Index (dRI), colorimetric assays, Fourier Transform InfraRed spectroscopy (FT-IR), Nuclear Magnetic Resonance (NMR), and compared to those of the crude fucoidan. After radiolabeling with ^99m^Tc according to a previously described method [18], the biodistribution of both fucoidans was assessed in healthy rats. SPECT imaging was then performed in a model of ischemia-reperfusion (I-R) to evaluate the efficiency of the purified fucoidan for *in vivo* detection of ischemic events in rat myocardium.

## 2. Results

### 2.1. Physico-Chemical Characterizations

#### 2.1.1. Molecular Weight Measurement

**Figure 1:**
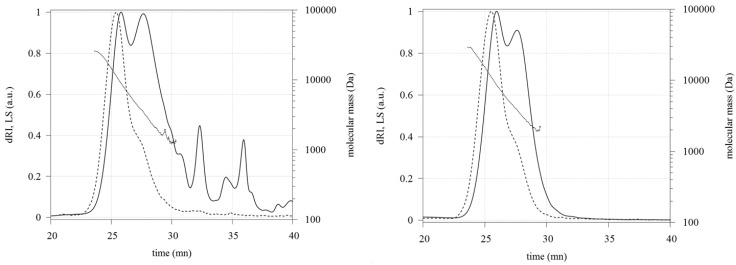
Refractive Index (plain line) and Light Scattering (dashed line) chromatograms and molecular mass profile (dotted line) of crude (Left) and purified (Right) fucoidans eluted in 0.1 M LiNO_3_ at 0.5 mL/min with Shodex SB-802.5 and SB-803 columns.

The purification treatment of crude fucoidan with aqueous calcium acetate led to a precipitate which was discarded by centrifugation. After dialysis and freeze-drying of the supernatant, purified fucoidan was obtained as a white fluffy powder in 45% average yield. The molecular weight measurements performed by HPSEC-MALLS-dRI showed two overlapping macromolecular populations (Figure 1): Light Scattering chromatograms showed one main peak with a shoulder at 27 min. The molecular weight distribution was linear from 24 to 28.5 min and ranged from 26 to 1 kDa. The respective peak masses were M P1 = 9.0 ± 0.1 kDa and M P2 = 3.5 ± 0.1 kDa for crude fucoidan and M P1 = 8.8 ± 0.1 kDa and M P2 = 4.0 ± 0.1 kDa for purified fucoidan. The Refractive Index chromatogram of purified fucoidan did not present peaks after 30 min, unlike crude fucoidan.

Average number (M_n_) and average weight (M_w_) molecular weights of purified fucoidan were increased compared to crude fucoidan but the polydispersity was not modified (Table 1), M_w_ being less affected by the purification, that is consistent with the elimination of small molecules by the purification process.

**Table 1:** Molecular weights of crude and purified fucoidans.

Fucoidans	*M_n_* (kDa)	*M_w_* (kDa)	*M_n_*/*M_w_*
Crude	3.5 ± 0.4	6.2 ± 0.3	1.5 ± 0.2
Purified	4.9 ± 0.2	7.5 ± 0.2	1.5 ± 0.1

#### 2.1.2. Chemical Composition 

l-fucose, uronic acid and sulfate contents assayed by colorimetric methods were gathered in Table 2. Crude fucoidan was mainly composed of l-fucose and sulfate with a lower amount of uronic acid. After the purification step with calcium acetate, purified fucoidan was enriched in l-fucose and sulfate by 71% and 16%, respectively. Moreover, the proportion of other components in fucoidan samples decreased from 37.2% to 15.2%.

**Table 2:** Composition of the fucoidan samples (w/w % of dried weight).

Fucoidans	l-Fucose	Uronic Acid	Sulfate	Other
Crude	25.0% ± 0.5%	16.1% ± 0.9%	21.7% ± 2.0%	37.2%
Purified	42.8% ± 2.2%	16.9% ± 0.4%	25.1% ± 0.8%	15.2%

#### 2.1.3. FTIR Analysis

**Figure 2:**
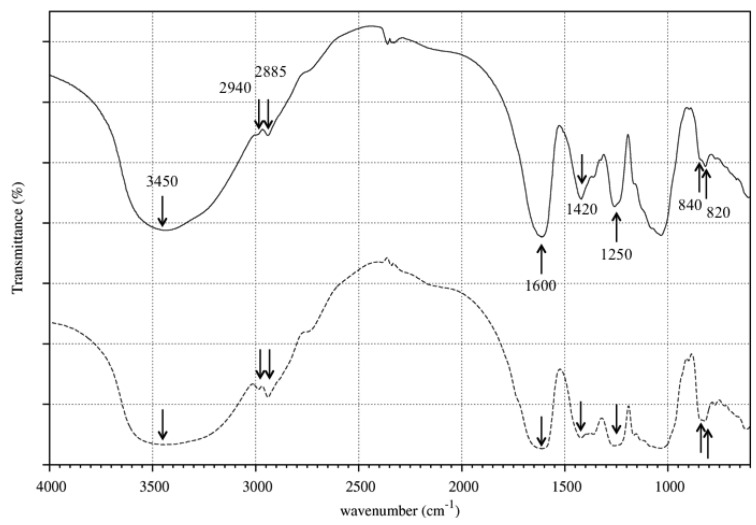
FT-IR spectra of crude (plain line) and purified (dashed line) fucoidans. Arrows indicate the characteristic bands for sulfated polysaccharides.

FT-IR spectra of crude and purified fucoidans (Figure 2) presented similar patterns corresponding to that of sulfated polysaccharides [21,22,23], a broad band at 3450 cm^−1^ (O-H groups stretching), two small sharp bands at 2940 cm^−1^ and 2885 cm^−1^ (C-H stretching of the pyranose ring and of the methyl group of fucose), two bands at 1600 cm^−1^ and 1420 cm^−1^ (COOH stretching vibration), a band around 1250 cm^−1^ (S=O stretching) and two small bands at 840 cm^−1^ and 820 cm^−1^ (axial and equatorial C-O-S bending, respectively).

#### 2.1.4. NMR Analysis

**Figure 3:**
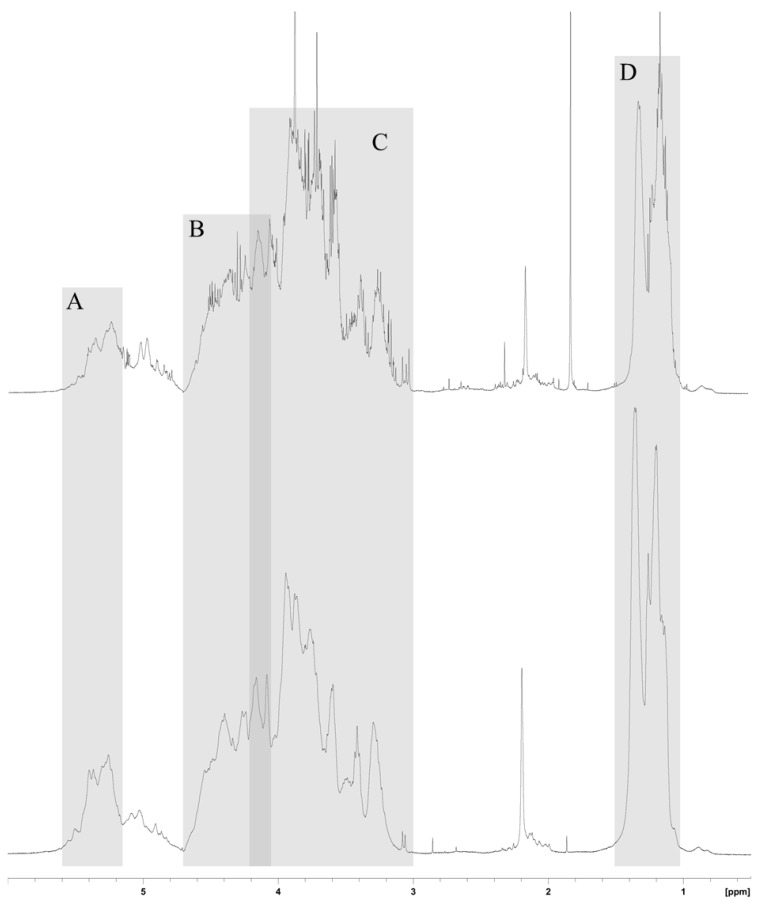
^1^H-NMR spectra (in 99.8% D_2_O, 500 MHz) of crude (upper) and purified (lower) fucoidans. (**A**) Anomeric protons from α-l-Fucose; (**B**) H-2, H-3 and H-4 of the sulfated fucose; (**C**) H-2, H-3, H-4 and H-5 of unsulfated fucose and uronic acid; (**D**) Methyl protons.

NMR spectra of both compounds also presented the same general features (Figure 3). Signals from anomeric protons H-1 arose from α-l-fucose residues and uronic acid residues between 5.6 and 5.15 ppm. H-2, H-3 and H-4 of the sulfated fucose could be localized between 4.66 and 4.10 ppm whereas from 4.20 to 3 ppm were H-2, H-3, H-4 and H-5 of unsulfated fucose and uronic acid. Lastly, the two peaks, around 1.2 and 1.35 ppm were assigned to the methyl group of fucose. A single peak at 2.2 ppm might be ascribed to acetyl groups.

### 2.2. In Vivo Studies of the ^99m^Tc-Radiolabeled Fucoidans

The radiolabeling of crude and purified fucoidans was performed with ^99m^Tc according to a previously described procedure [18]. The radiochemical purity was superior to 95% for both fucoidans without any significant difference.

#### 2.2.1. Biodistribution Studies of the Radiolabeled Fucoidans

**Figure 4:**
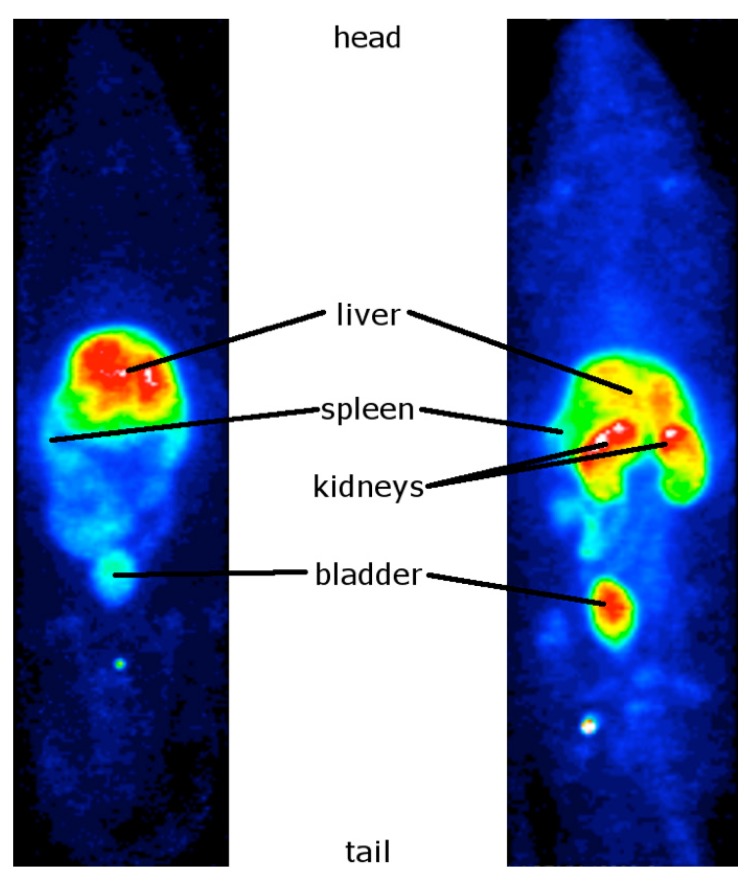
Representative whole body SPECT images of the ^99m^Tc-radiolabeled fucoidans: crude (left) and purified (right). The rats (five for crude fucoidan and four for purified fucoidan) were imaged 2 h after injection of 2 µg of the samples.

**Figure 5:**
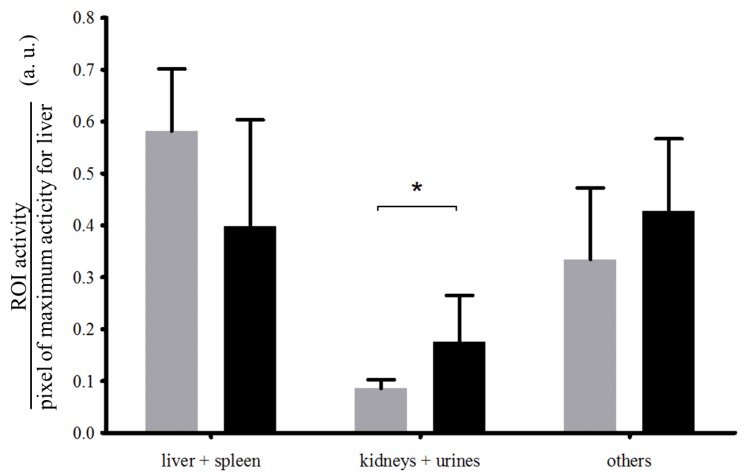
Quantification of the biodistribution of crude fucoidan (grey) and purified fucoidan (black) based on the whole body SPECT images obtained 2 h after *i.v.* injection of the ^99m^Tc-radiolabeled fucoidans in rats (respectively *n* = 5 and *n* = 4) expressed as mean ± SD. Three Regions of Interest (ROI) were assessed: the hepatosplenic system (liver + spleen), the urinary system (kidneys + bladder) and the other parts (the hepatosplenic system and the urinary system activities subtracted from whole body activity). * *p* < 0.05.

The uptake of ^99m^Tc-crude fucoidan was intense in the rat liver and only faint in the bladder and kidneys (Figure 4, left panel). In contrast, the uptake of purified fucoidan was faint in the liver and intense in both bladder and kidneys (Figure 4, right panel). Quantifications of uptake ratios obtained by SPECT are presented in Figure 5. No free ^99m^Tc was detected in thyroid for both compounds indicating a stable complexation even after 2 h *in vivo*. Note that the background signal of the body was higher for purified fucoidan than for crude fucoidan since the images were normalized on the highest pixel signal (in white on the color images).

#### 2.2.2. Myocardial Ischemia-Reperfusion

**Figure 6:**
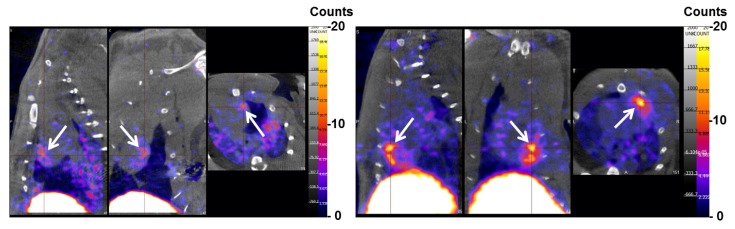
SPECT/CT (from left to right: sagittal, coronal and axial plane) of myocardial ischemia-reperfusion in rat, acquired 2 h after the injection of the radiolabeled crude fucoidan (**A**) and purified fucoidan (**B**). The SPECT color-scale was normalized (max: 20 counts/pixel). The focal uptake of the tracer was localized in the area of ischemia provoked in the left ventricle (white arrow), with a myocardium to background (blood pool) ratio of 1.9 with the crude fucoidan and 3.3 with the purified form.

**Figure 7:**
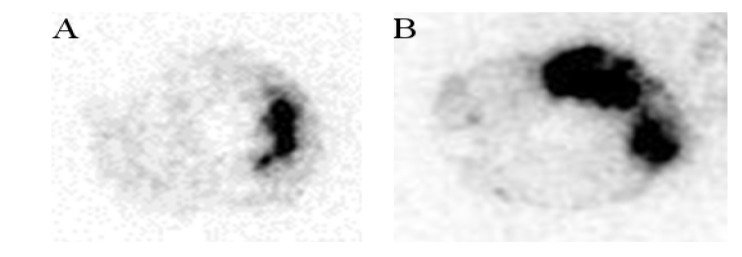
Autoradiography (20 µm thickness transverse slices) of the myocardium after injection of the radiolabeled crude fucoidan (**A**) and purified fucoidan (**B**) in an I-R rat model. The uptake of the radiolabeled fucoidans appears in black.

In the model of myocardial Ischemia-Reperfusion in rat, purified fucoidan facilitated the *in vivo* detection of the ischemic myocardium with a signal intensity twice that of the crude form (Figure 6). A similar finding was obtained by autoradiography (Figure 7). The ratio of the injured tissue (dark area) activity over the normal myocardium was computed for crude fucoidan (4.9 ± 0.2) and for purified fucoidan (8.7 ± 2.1).

## 3. Discussions

Fucoidans are interesting bioactive sulfated polysaccharides to be developed for clinical purposes. They are endowed with numerous biological activities, are cheap and their preparations from marine algae allow protocols compatible with industrial constraints without costly specific equipment. However, there is currently no standard method to obtain a bioactive well defined fucoidan from crude carbohydrate extracts. Fucoidans are always obtained as mixtures of macromolecular species, the structures of which depend on many parameters, such as the algal species or the period and location of harvesting and the purification methods [24,25]. Commercial products with the same identification are for instance highly variable in their molecular weight (from 20 to 170 kDa) and composition.

Numerous studies have been published on structure-activity relationships of fucoidans from marine algae [12,26] since this species was identified by Kylin one century ago [27]. Fucoidans belong to the family of fucans including two other carbohydrate species: ascophyllans and sargassans [28]. Fucoidans refer to polysulfated poly-l-fucose, although xylose, glucose, galactose or glucuronic acids can be associated [29], as ascophyllans and sargassans are uronic acid-rich and galactose-rich polysaccharidic species, respectively. Uronic acids are frequently recovered in fucoidans from algae either as co-extracted polysaccharidic structures, or as discrete short branchings [25]. Eventually, repeated simple fucoidan structures can be obtained but always after several purification steps from crude fucoidan extracts [30,31,32,33]. To date, only sulfated fucose rich species have been reported to be responsible for the biological properties of fucoidans [11,12]. Recent studies demonstrated that Ascophysicent^®^ (crude fucoidan), a commercially available low molecular weight fucoidan extracted from *Ascophyllum nodosum*, is a relevant biomarker for the molecular imaging of thrombus and vascular activation in atherothrombotic diseases [18,19]. However, sulfate and fucose represent less than 50 percent of the dry weight of crude fucoidan with a remaining high proportion of uronic acids and other sugars. It is likely that it contains a mixture of bioactive sulfated fucose-based polymers and some polyuronic acids, likely ascophyllan. This work aimed at (i) purifying crude fucoidan by precipitating the polyuronic structures with calcium ions; (ii) compare biodistribution of crude *vs.* purified fucoidan with regards to the route of blood clearance and (iii) demonstrate that the purification process did not alter radiolabeling efficiency and *in vivo* binding.

The purification with calcium ions was intended to remove uronic acid chains contained in the crude fucoidan. As the alginates are expected to be removed by the industrial extraction process (data not shown), it has been thought that the uronic acids came from the ascophyllans, a sulfated polysaccharide with an uronic acid backbone and sulfated polyfucose branchings [34,35], that could be co-extracted with the fucoidan. The uronic acid backbone of ascophyllans would form complexes with calcium ions and then precipitate. Our findings showed that the FT-IR and ^1^H-NMR spectra of crude and purified fucoidans presented typical features of a sulfated polysaccharide with sulfation at O-2, O-3 and O-4 positions of l-fucose as previously demonstrated with fucoidan extracts from *A. nodosum* [22,36]. Compared to other fucoidans extracted from *A. nodosum*, crude fucoidan is composed of two low molecular weight species: one with a lower fucose rate and the other with a higher uronic acid rate [29,37,38]. By comparing the quantity of uronic acids to the total quantity of fucose and sulfate groups of crude and purified fucoidans—34.5% and 24.9%, respectively—it appeared that around 10% of uronic acids were removed by the calcium treatment. Moreover, the molecular weight distribution of purified fucoidan was shifted to slightly higher molecular weights as small molecules were removed.

The radiolabeling with ^99m^Tc was achieved for both species with a radiochemical purity higher than 95% similar to that of the ^99m^Tc-heparin [39]. *In vivo*, ^99m^Tc-crude fucoidan was localized in part in the liver and the spleen which are the targeted organs of the reticulo-endothelial system (RES). This *in vivo* data is interesting since low molecular weight fucoidan from *A. nodosum* was reported to have *in vitro* anti-complement activity [16]. The higher accumulation of ^99m^Tc-crude fucoidan in the liver and spleen compared to that of ^99m^Tc-purified fucoidan could be due to the higher quantity of uronic acids.

Finally, the purification procedure did not affect *in vivo* binding of fucoidan to endothelial cells activated by a transient ischemic event. In the Ischemic-Reperfusion rat model, focal uptakes of the polysaccharides were detectable for both ^99m^Tc-crude fucoidan and ^99m^Tc-purified fucoidan. Interestingly, it was more intense for the purified fucoidan and the autoradiographies strengthened this observation.

## 4. Experimental Section

### 4.1. Polysaccharide and Common Chemicals

Fucoidan was obtained from Algues & Mer (Ascophycient^®^, batch #ASPHY12399, Ouessant, France), DEAE-cellulose resin from Sigma Aldrich and dialysis membrane from Spectra/Por, (MWCO 1 kDa, Dominique Dutscher, France). Common chemicals were purchased in analytical grade from Carlo Erba, Sigma-Aldrich and Fisher Scientific and used without further purifications.

### 4.2. Molecular Weight Determination

The absolute average molecular weights and molecular weight distributions were determined at room temperature by coupling online a high performance size exclusion chromatograph (HPSEC), a multi-angle laser light scattering detector (MALLS), a viscometer and a differential refractive index (dRI) detector. 0.1 M LiNO3, used as carrier, was filtered through a 0.1 µm filter unit (Millipore, Billerica, USA), carefully degassed (DGU-20A3R Shimadzu, Kyoto, Japan), and eluted at a 0.5 mL/min flow rate (LC10Ai Shimadzu, Kyoto, Japan). 100 µL of a 0.45 µm-filtered sample solution (at about 20 mg/mL) were injected with an automatic injector (SIL-20A HT Shimadzu, Kyoto, Japan). The SEC line consisted of an OHpak SB-G guard column for protection and two OHpak SB-802.5 and-803 HQ columns (Showa Denko Europe, Munich, Germany) in series. The column packing was a poly (hydroxymethacrylate) gel. The MALLS photometer, a DAWN HELEOS II from Wyatt Technology Inc. (Santa Barbara, CA, USA) was provided with a fused silica cell and a Ga-As laser (λ = 665.8 nm). The viscometer was a ViscoStar II from Wyatt Technology Inc. (Santa Barbara, CA, USA). The whole collected data: light scattering (LS), dRI and viscosity were analyzed using the Astra v6.0.6 software package. Molar mass were obtained with the Zimm order 1 method using angles between 53.1° and 140°. The concentration of each eluted fraction was determined with dRI (RID10A Shimadzu, Kyoto, Japan) according to the measured values of dn/dc (0.144 mL/g). The determination of the average intrinsic viscosity allowed us to obtain the average hydrodynamic volume (Vh, *i.e.*, the average hydrodynamic radius Rh) using the Einstein-Simha equation: $V_{h}=\frac{\left[\eta \right]\cdot M}{\nu \cdot N_{A}}$ where N_A_ is Avogadro’s number, M is the molar mass, [η] is the intrinsic viscosity (g/mL), and ν is a conformational parameter that takes the value of 2.5 (for spherical random coil).

### 4.3. Purification of Fucoidan

The purification of fucoidan was achieved after dissolution (160 mg/mL) of crude fucoidan with aqueous calcium acetate (20 mM) during 4 h at 55 °C under magnetic stirring and the pH was constantly adjusted between 6.5 and 7.5. The solution was left at 4 °C overnight and then centrifuged 15 min at 4500 rpm (Allegra X-15R centrifuge, Beckman-Coulter France, Villepinte, France). The supernatant was dialyzed against water (MWCO 1 kDa, Dominique Dutscher, France) and freeze-dried.

### 4.4. Colorimetric Assay

The colorimetric assay was achieved on microplate and ELx800 Absorbance Microplate Reader (BioTek, Colmar, France) and processed with Gen-5 software (BioTek, Colmar, France).

#### 4.4.1. Fucose

The fucose rate determination was adapted from the Dische method [40]. In a 96-wells plate, 50 µL of sample (about 0.3 mg/mL) was mixed with 200 µL of sulfuric acid (18 M). The plate was heated during 30 min at 80 °C then left at room temperature for cooling down. 8 µL of l-cysteine hydrochloride at 3% (w/v) was added and left to react for 1 h at 4 °C. Then absorbance was computed as follows: OD_fucose_ = [OD_405 _(sample) − OD_405 _(blank)] − [OD_450 _(sample) − OD_450 _(blank)]. The amount of fucose was determined from a standard curve obtained with l-fucose solutions (25–500 µg/mL).

#### 4.4.2. Glucuronic Acid

Glucuronic acid rate determination was adapted from Bitter *et al.* [41]. In a 96-wells plate, 35 µL of sample (about 0.3 mg/mL) was mixed with 200 µL of sodium tetraborate (0.025 M) in sulfuric acid (18 M). The plate was heated during 30 min at 80 °C then left at room temperature for cooling down. 14 µL of carbazol solution at 0.15% (w/v) in ethanol was added and heated 1 h at 80 °C, and then the absorbance was read at 540 nm. The amount of uronic acid was determined from a standard curve obtained with d-glucuronic acid solutions submitted to the same process.

#### 4.4.3. Sulfate

Sulfate rate was obtained by formation of methylene blue after acidic hydrolysis of the samples, reduction of sulfate as hydrogen sulfide (H_2_S), and formation of methylene blue from *N,N*-dimethyl phenylene diamine dihydrochloride in strong acidic medium in presence of ferric chloride. Conditions were optimized from Gustafsson [42] and Kuban *et al.* [43]. Briefly, 100–200 µL of the sample were added to 5 mL of a reducing mixture prepared with 100 mL of concentrated hydroiodic acid, 25 mL of glacial acetic acid and 2.5 g of sodium hypophosphite. The mixture was refluxed during 20 min through a water-cooled condenser under a stream of N_2_ (100 mL/min) which carried away evolved H_2_S. After bubbling through a gas-washing column (20 mL of Tris buffer 0.1 M, pH 7.2), H_2_S was trapped as zinc sulfide (ZnS) in 30 mL of a solution of zinc acetate prepared by diluting 5 mL at 0.5 M and sodium acetate 0.1 M with 25 mL of deionized water. Eight mL of 16 mM ferric chloride in H_2_SO_4_, 0.1 M, and 2 mL of 3.7 mM *N,N*-dimethyl-phenylene-diamine dihydrochloride in H_2_SO_4_, 9 M were added to the ZnS solution, and the final volume was adjusted to 50 mL with deionized water. The vial was maintained at room temperature in the dark for 20 min and the absorption was measured at 665 nm with a UV-visible spectrophotometer (Lambda 12, Perkin-Elmer, Courtaboeuf, France). The amount of sulfur was determined from a standard curve obtained with potassium sulfate solutions submitted to the overall process. This method did not require a special treatment of the samples and was not sensitive to ferric ions contrary to sulfur elemental analysis or turbidimetric assays.

### 4.5. Infrared Spectra

Infrared spectra were acquired between 400 and 4000 cm^−1^ with an AVATAR 370 FT-IR spectrometer (Thermo-Nicolet, Villebon, France) with 32 scans/sample and a resolution of 2 cm^−1^. Dry samples were pressed with potassium bromide (2% w/w). Spectra were processed and analyzed with OMNIC v6.1 (Thermo-Nicolet software).

### 4.6. Nuclear Magnetic Resonance (NMR)

All NMR experiments were conducted on a Bruker AVANCE III spectrometer (BioSpin Bruker, Wissembourg, France) operating at a proton frequency of 500 MHz with a 5 mm gradient indirect detection probe, at a probe temperature of 300 K. The samples were exchanged twice with 99.8% D_2_O with intermediate freeze drying and dissolved in 0.6 mL of 99.96% D_2_O. 1-D proton spectra were acquired with 16 scans and 32 K data points with a spectral width of 5000 Hz. Typical ^1^H 9.5 µs-pulse length and relaxation delay of 1 s were used. Water signal was suppressed by a presaturation sequence at the water signal frequency.

### 4.7. Radiolabeling Procedure

The radiolabeling of fucoidan has been performed according to the general process of reduction of pertechnetate (TcO_4_^−^) by stannous ions in order to obtain a complex between a reduced form of ^99m^Tc and the ligand. The procedure is the most widely used to produce ^99m^Tc-labeled radiopharmaceuticals both in the preclinical and clinical domains [44]. The mechanism of complexation generally accepted involves the formation of positively charged reduced forms of ^99m^Tc [Tc(V) and/or Tc(III)] that will react with negatively charged domains of the ligand [44]. This procedure has already been applied to radiolabeling of polysaccharides such as heparin [45] and dextran [46]. Briefly, ^99m^Tc was eluted from a ^99^Mo/^99m^Tc generator as an isotonic solution with high specific activity. The sodium pertechnetate (^99m^TcO_4_Na, between 300 and 400 MBq in <200 µL saline, freshly eluted), 1 µg stannous chloride (1 µg/µL; Sigma-Aldrich, Saint-Quentin Fallavier, France) and 1 µg potassium borohydride (1 µg/µL; Sigma-Aldrich, Saint-Quentin Fallavier, France) were added to a vial containing 10 µg of fucoidan (1 mg/mL), and left to incubate during 1 h at room temperature. The quality control was performed with instant thin-layer chromatography (ITLC) developed in methyl-ethyl-ketone buffer. The radiochemical purity was always superior to 95%. No supplementary purification was performed on the radiolabeled fucoidan.

### 4.8. Biodistribution and Experimental Model of Myocardial Ischemia-Reperfusion

For both the biodistribution and ischemia-reperfusion rat model, each rat was injected with 200–500 µL of 60–74 MBq of radiolabeled fucoidan, corresponding to about 2 µg of fucoidan and the injected dose pH was neutral (7 and 7.5). The rats weighted between 310 and 350 g.

Biodistribution of radiolabeled fucoidans was performed in healthy male Wistar rats (Janvier, France) in order to determine the preferential route of blood clearance after intravenous injection. Since we previously determined that blood clearance of fucoidan was maximal during the first 2 h after administration [18], we set the delay between injection and biodistribution assessment at 2 h. We determined *in vivo* biodistribution of fucoidans by SPECT imaging which is inherently quantitative and is now widely used for this purpose.

Whole body acquisitions were performed under isoflurane anesthesia after intravenous injection (penile vein) of 50 MBq of crude (*n* = 5) or purified (*n* = 4) fucoidans. Total body activity was estimated using a volume of interest encompassing the whole field of view, subtracted from residual activity remaining at the injection site (penis). Then, relative organ retention (defined for liver, spleen, left kidney, and bladder) was calculated as the ratio of activity within 3-dimensional volumes of interest over total body activity. Results were then displayed as renal excretion (kidney × 2 + bladder) and reticulo-endothelial retention (liver + spleen).

The model of myocardial ischemia-reperfusion was performed in four male Wistar rats. The proximal left anterior descending coronary artery was occluded using a suture around a catheter for 20 min, then was reperfused by cutting the suture along the catheter in rats under general anesthesia (ketamine/xylazine) and positive pressure ventilation. Injection of the radiotracer was performed after 2 h of reperfusion. This study was conducted under authorization of the French Direction of the Veterinary Services (No. 2012-15/698-106).

### 4.9. Imaging Procedures

#### 4.9.1. Acquisition and Reconstruction Parameters

Imaging was performed 2 h after intravenous injection of radiolabeled fucoidans. Helical SPECT/CT scans were performed under isoflurane anesthesia using a 4-headed multiplexing multipinhole camera (NanoSPECT/CT plus, Bioscan Inc., Washington, DC, USA). Each head was equipped with a tungsten collimator (Rat Whole Body—High sensitivity). Flat-panel detector CT was performed first (tube voltage: 55 kV, tube current: 145 mAs), then whole body SPECT acquisition was performed with the following parameters: helical scan with 28 projections/rotation plus circular scan at the beginning and at the end of the scan range, matrix size: 256 × 256, zoom: 1.14 (pixel size: 1 mm^2^). SPECT data were reconstructed using the HiSPECT (Bioscan Inc., Washington, DC, USA) iterative reconstruction software on a dedicated workstation, and visualized using InVivoScope software with co-registration of SPECT and CT images. 

#### 4.9.2. Data Analysis

Radiolabeled fucoidan biodistribution in healthy rats was carried out in order to establish evidence of the preferential route of blood clearance after intravenous injection. Total body activity was estimated using a volume of interest encompassing the whole field of view, subtracted from residual activity remaining at the injection site (penis). Then, relative organ retention (defined for liver, spleen, left kidney, and bladder) was calculated as the ratio of activity within 3-dimensional volumes of interest over total body activity. Results were then displayed as renal excretion (kidney × 2 + bladder) and reticulo-endothelial retention (liver + spleen).

Ischemia-reperfusion model scintigrams were assessed visually to determine the presence of a focal tracer uptake in the myocardium.

Autoradiography: after completion of SPECT/CT, animals were euthanized with pentobarbital overdose. Tissue samples were frozen and cut into transverse sections of 20 µm thickness which were exposed in a digital β-imager (Beta Imager, Biospace Lab, Paris, France) for 6 h. Quantification was performed by calculating the ratio between the activity (mean counts/mm^2^) of the area at risk and the activity of a region of interest drawn over normal myocardium.

### 4.10. Statistical Analyses

Statistics were computed with GraphPad Prism v.5 software. Chemical compositions of the samples were compared with a two-tailed *t*-test where *p* < 0.05 was considered significant. For biodistribution, each sample was compared 2 by 2 in a ROI using a two-tailed Mann-Whitney test and was considered significant at *p* < 0.05.

## 5. Conclusions

Ascophyscient^®^ is a commercially available product extracted from Ascophyllum nodosum mainly composed of fucose, sulfate and uronic acids. After a simple purification step, its composition was enriched in sulfated fucose. The biodistribution of the purified fucoidan in healthy rats was improved ensuring that the renal elimination was favored. Finally, the binding of ^99m^Tc-purified fucoidan to the activated endothelium was validated in rat model of myocardial ischemia. As a low cost and easily labeling ligand of ischemic events, this purified fucoidan is promising for the clinical development of a molecular imaging probe for myocardial infarctions.
